# The origin of esterase activity of Parkinson's disease causative factor DJ-1 implied by evolutionary trace analysis of its prokaryotic homolog HchA

**DOI:** 10.1016/j.jbc.2024.107476

**Published:** 2024-06-13

**Authors:** Aiko Watanabe, Fumika Koyano, Kenichiro Imai, Yohei Hizukuri, Shizuka Ogiwara, Tomoya Ito, Jun Miyamoto, Chihiro Shibuya, Mayumi Kimura, Kazuya Toriumi, Chie Motono, Makoto Arai, Keiji Tanaka, Yoshinori Akiyama, Koji Yamano, Noriyuki Matsuda

**Affiliations:** 1 Division of Advanced Pathophysiological Science, Department of Biomolecular Pathogenesis, Medical Research Institute, Tokyo Medical and Dental University, Bunkyo, Tokyo, Japan; 2 Cellular and Molecular Biotechnology Research Institute, National Institute of Advanced Industrial Science and Technology (AIST), Koto, Tokyo, Japan; 3 Global Research and Development Center for Business by Quantum-AI Technology (G-QuAT), National Institute of Advanced Industrial Science and Technology (AIST), Tsukuba, Ibaraki, Japan; 4 Laboratory of Biological Membrane System, Institute for Life and Medical Sciences, Kyoto University, Kyoto, Japan; 5 Schizophrenia Research Project, Tokyo Metropolitan Institute of Medical Science, Setagaya, Tokyo, Japan; 6 Computational Bio Big-Data Open Innovation Laboratory (CBBD-OIL), National Institute of Advanced Industrial Science and Technology (AIST), Waseda University, Shinjuku, Tokyo, Japan; 7 Laboratory of Protein Metabolism, Tokyo Metropolitan Institute of Medical Science, Setagaya, Tokyo, Japan

**Keywords:** Parkinson disease (PD), PARK7, DJ-1, oxo-aldehyde, hydratase, esterase, prokaryote, catalytic triad

## Abstract

DJ-1, a causative gene for hereditary recessive Parkinsonism, is evolutionarily conserved across eukaryotes and prokaryotes. Structural analyses of DJ-1 and its homologs suggested the 106th Cys is a nucleophilic cysteine functioning as the catalytic center of hydratase or hydrolase activity. Indeed, DJ-1 and its homologs can convert highly electrophilic α-oxoaldehydes such as methylglyoxal into α-hydroxy acids as hydratase *in vitro*, and oxidation-dependent ester hydrolase (esterase) activity has also been reported for DJ-1. The mechanism underlying such plural activities, however, has not been fully characterized. To address this knowledge gap, we conducted a series of biochemical assays assessing the enzymatic activity of DJ-1 and its homologs. We found no evidence for esterase activity in any of the *Escherichia coli* DJ-1 homologs. Furthermore, contrary to previous reports, we found that oxidation inactivated rather than facilitated DJ-1 esterase activity. The *E. coli* DJ-1 homolog HchA possesses phenylglyoxalase and methylglyoxalase activities but lacks esterase activity. Since evolutionary trace analysis identified the 186th H as a candidate residue involved in functional differentiation between HchA and DJ-1, we focused on H186 of HchA and found that an esterase activity was acquired by H186A mutation. Introduction of reverse mutations into the equivalent position in DJ-1 (A107H) selectively eliminated its esterase activity without compromising α-oxoaldehyde hydratase activity. The obtained results suggest that differences in the amino acid sequences near the active site contributed to acquisition of esterase activity *in vitro* and provide an important clue to the origin and significance of DJ-1 esterase activity.

*DJ-1/PARK7* is a causative gene for recessive familial Parkinson disease (hereditary Parkinsonism) (1) and is thought to function as an antioxidative stress protein that plays an important role in protecting cells from reactive oxygen species and mitochondrial damage (2, 3, 4). Different and occasionally inconsistent molecular functions, including as a molecular chaperone (5, 6), a transcriptional regulator (7, 8), and a protease (9) have been ascribed to DJ-1 (10). Very recently, DJ-1 was shown to prevent phosphoglycerate modifications that are derived from reactive derivative of 1,3-phosphoglycerate (cyclic-1,3-phosphoglycerate) (11). Despite these reports, its biochemical function has not been fully characterized. The absence of diversity in the DJ-1 molecular structure (12, 13, 14, 15), however, can provide a useful starting point for addressing this knowledge gap. DJ-1 belongs to the well-conserved PfpI/Hsp31/DJ-1 superfamily, members of which are characterized by a conserved cysteine in a nucleophilic elbow structure that resembles cysteine proteases (12, 13, 14, 15). This strongly suggests that DJ-1 is an enzyme and that the nucleophilic cysteine (C106) plays a critical role in the enzymatic reaction.

From this perspective, the reports of DJ-1 functioning as a deglycase, glyoxalase, or esterase are intriguing. While DJ-1 deglycase activity is likely derived from its glyoxalase activity acting on free methylglyoxal present in fast equilibrium with hemithioacetals and hemiaminals (16, 17, 18, 19), there are reports of DJ-1 glyoxalase and esterase activities with C106 as the active center (20). *In vitro* studies have shown that DJ-1 possesses α-oxoaldehyde hydratase activity, namely DJ-1 converts α-oxoaldehydes such as methylglyoxal into α-hydroxy acids such as lactate, an activity referred to as glyoxalase III (21, 22). DJ-1 esterase activity has been evaluated with a colorimetric assay using 4-nitrophenyl acetate (pNPA) as a model substrate (23). Further, *in vitro* assays of both the α-oxoaldehyde hydratase and esterase activities have facilitated the identification and analysis of DJ-1 inhibitors (24, 25, 26).

The DJ-1 esterase activity, however, is unconventional as it was reported to be oxidation-dependent (23). In that study, esterase functionality was activated rather than inactivated in response to replacing the active site C106 with Asp or H_2_O_2_-mediated oxidation of C106 (23). The hypothesis that DJ-1 is activated in response to oxidation is intriguing since DJ-1 has been reported to function as an antioxidative stress protein, and DJ-1 overproduction resulted in increased cellular resistance to oxidative stress (4, 27, 28). However, no molecular reaction model has been proposed to explain this atypical enzymatic function and no additional supporting evidence has been reported.

Although it is well-accepted that DJ-1 can function as an esterase *in vitro*, we sought to re-evaluate this atypical oxidation-dependent activation mechanism. Since DJ-1 is highly conserved in eukaryotes and prokaryotes like *Escherichia coli* (29, 30), it is reasonable to expect that esterase activity is also evolutionarily conserved if it is an essential function of DJ-1 family proteins. However, it is entirely unclear if this is indeed the case. In this study, we assayed the esterase activity of the *E. coli* DJ-1 homologs YajL, YhbO, HchA, and ElbB. Systematic mutational analysis of HchA, the first identified prokaryotic DJ-1 homolog with α-oxoaldehyde hydratase activity (31), provided unexpected insights into esterase functionality. These results provide a deeper, more nuanced understanding of the mechanism underlying DJ-1 esterase activity and its evolutionary origins.

## Results

**Figure 1:**
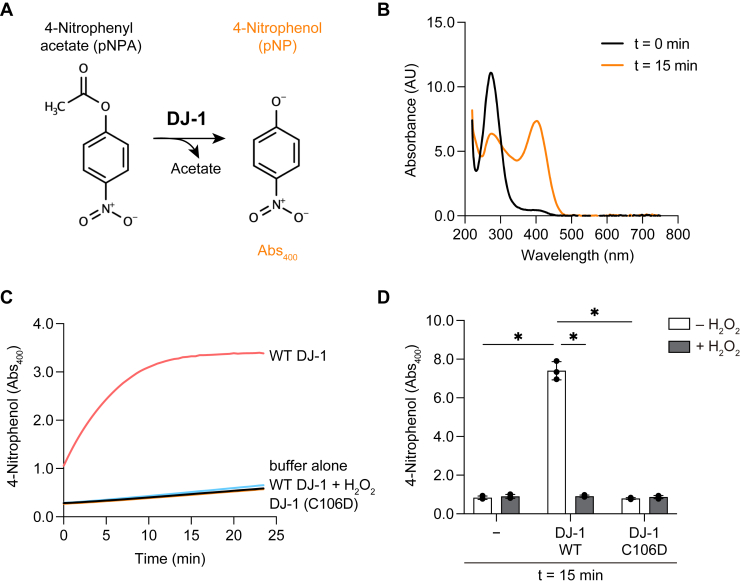
**C106 oxidation and oxidation-mimetic mutation inhibit DJ-1 esterase activity.***A*, DJ-1 esterase reaction mechanism for 4-nitrophenol (pNP) formation. DJ-1 conversion of 4-nitrophenyl acetate (pNPA) to acetate and pNP was monitored *via* an increase in *A*_400_ by the pNP reaction product. *B*, DJ-1–mediated generation of pNP from pNPA with the characteristic *A*_400_. Absorbance spectra were monitored before (*black*) or 15 min after (*orange*) the start of the reaction. *C*, pNP generation over time. pNPA was incubated with the indicated proteins as described in the experimental procedures. WT DJ-1 was preincubated with 100 μM H_2_O_2_ for 1 h at room temperature prior to being mixed with pNPA. Representative data for three individual experiments are shown. *D*, *A*_400_ of pNP after 15 min incubation with or without WT DJ-1 or the C106D mutant ± H_2_O_2_ treatment (all n = 3). *Bars* represent the mean ± SD of three experiments. ∗*p* < 0.05 using two-way ANOVA with Bonferroni’s multiple comparisons test (*D*).

We first determined if DJ-1 exhibits oxidation-activated esterase activity as reported previously (23). The esterase activity was assessed using a colorimetric assay with pNPA as the substrate. When pNPA is hydrolyzed and converted into 4-nitrophenol (pNP) and acetate, there is a concomitant increase in pNP absorbance at 400 nm (*A*_400_). Therefore, *A*_400_ can be used to monitor DJ-1 esterase activity (Fig. 1*A*), which we confirmed (Fig. 1*B*) as reported previously (23, 24). We next investigated the atypical oxidation-dependent activation of the esterase. Oxidation-mimetic mutations at the active center (C106D) and pretreatment of DJ-1 with H_2_O_2_ were reported to accelerate ester hydrolysis (23). However, no esterase activity was observed with the purified DJ-1 C106D mutant (Fig. 1, *C* and *D*). Similarly, pretreatment of DJ-1 with H_2_O_2_ resulted in a complete loss of esterase activity rather than an increase in activity (Fig. 1, *C* and *D*). These results are consistent with DJ-1 functioning as a conventional esterase that is inactivated by Cys oxidation, and not as an atypical esterase as reported (23).

**Figure 2:**
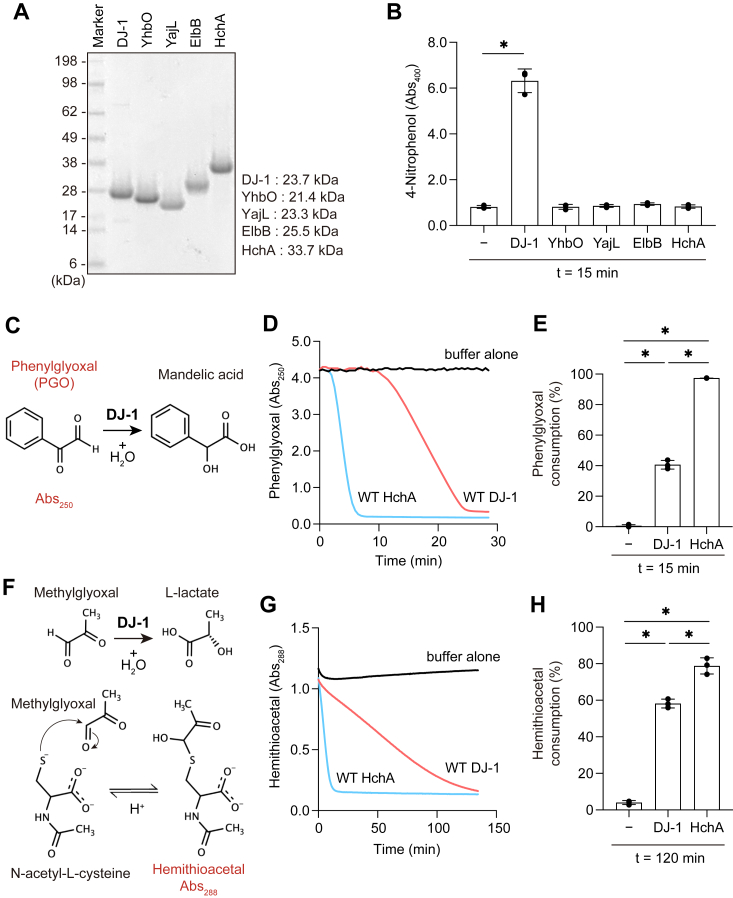
**HchA possesses phenylglyoxalase and methylglyoxalase activities but not esterase activity.***A*, Coomassie-stained protein gel of recombinant DJ-1 homologs after purification. Gel lanes were loaded with 5 μg of recombinant DJ-1 or one of the *Escherichia coli* DJ-1 homologs. DJ-1 (lane 1), YhbO (lane 2), YajL (lane 3), ElbB (lane 4), and HchA (lane 5). The calculated molecular sizes of the recombinant proteins are indicated to the right of the gel. *B*, pNP generation from pNPA was observed with DJ-1 but not the bacterial DJ-1 homologs. DJ-1, EhbO, YajL, ElbB, or HchA protein (1 μM) was incubated with 1.6 mM pNPA for 15 min (all n = 3). *C*, the phenylglyoxal (PGO) hydratase reaction mechanism. PGO conversion into mandelic acid by DJ-1 can be monitored by a reduction in *A*_250_. *D*, PGO *A*_250_ in the presence of DJ-1 (*red*) or HchA (*blue*) over time at 37 °C. PGO was incubated with DJ-1 or HchA as described in the experimental procedures. The *black line* corresponds to buffer alone. *E*, percentage PGO consumption after 15 min incubation with either DJ-1 or HchA (all n = 3). PGO consumption values were calculated based on the difference in *A*_250_ before and after the start of the reaction. *F*, the methylglyoxal hydratase reaction mechanism. *A*_288_ increases following hemithioacetal formation from methylglyoxal and N-acetyl-cysteine. DJ-1 catalysis of methylglyoxal shifts the equilibrium reaction away from hemithioacetal formation with a concomitant reduction in *A*_288_. *G*, hemithioacetal *A*_288_ in the presence of DJ-1 (*red*) or HchA (*blue*) at 37 °C. Hemithioacetal was incubated with DJ-1 or HchA as described in the Experimental procedures. The *black line* corresponds to buffer alone. *H*, hemithioacetal *A*_288_ ratio following 120 min incubation with buffer alone, DJ-1, or HchA (all n = 3). Data shown in *D* and *G* are representative of three individual experiments. Data in *B*, *E*, and *H* are the mean ± SD of three experiments. ∗*p* < 0.05 using one-way ANOVA with Dunnett’s multiple comparisons test (*B*, *E*, and *H*).

**Figure 3:**
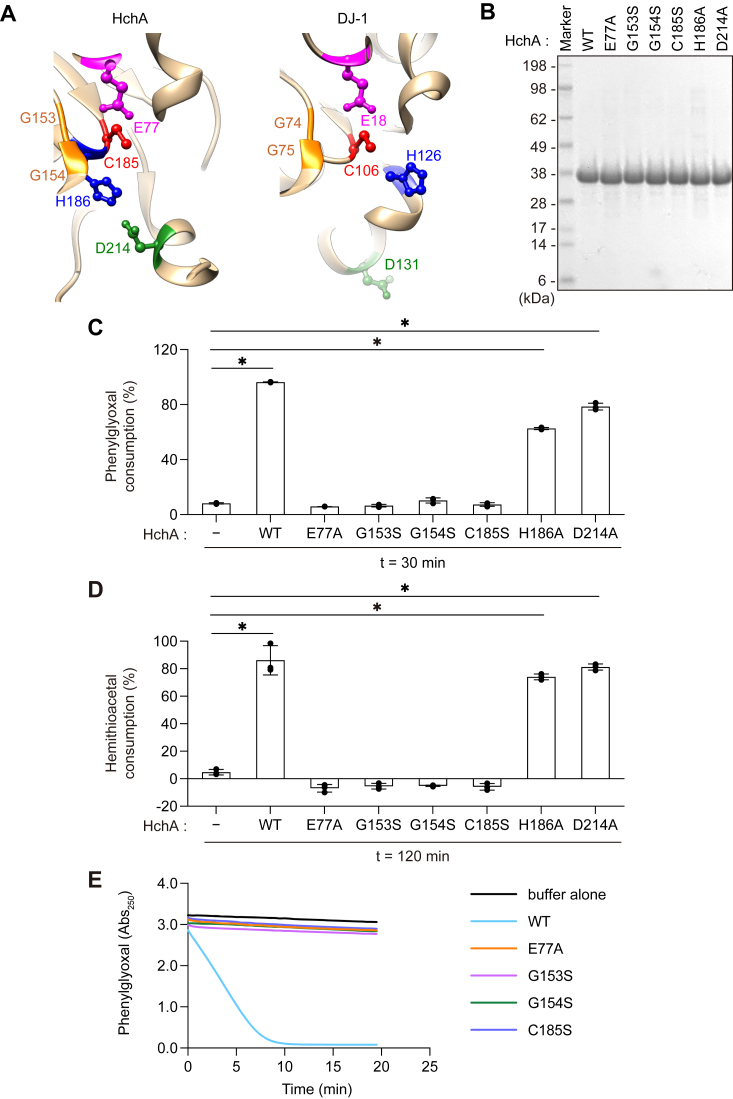
**HchA α-oxoaldehyde hydratase activity was abolished by E77A, G153S, G154S, and C185S mutations.***A*, structure of the HchA (*left*) and DJ-1 (*right*) catalytic cores. Equivalent residues between HchA and DJ-1 are highlighted by the same color. The HchA residues selected for mutation are E77 (*magenta*), G153 (*orange*), G154 (*orange*), C185 (*red*), H186 (*blue*), and D214 (*green*). *B*, Coomassie-stained protein gel of recombinant HchA mutants after purification. Gel lanes were loaded with 5 μg of HchA WT (lane 2), E77A (lane 3), G153S (lane 4), G154S (lane 5), C185S (lane 7), H186A (lane 7), and D214A (lane 8). *C*, PGO consumption after 30 min incubation with buffer alone, WT HchA, or the indicated HchA mutant proteins as described in the Experimental procedures. PGO consumption values were calculated based on the difference in *A*_250_ before and after the start of the reaction (n = 3). *D*, hemithioacetal consumption by WT HchA or the indicated mutants. The hemithioacetal reaction period was 120 min as described in the Experimental procedures (all n = 3). *E*, PGO *A*_250_ over time. *Lines* correspond to WT HchA (*cyan*) or HchA with E77A (*orange*), G153S (*magenta*), G154S (*green*), or C185S (*dark blue*) mutations. Representative data from three individual experiments are shown. Data in *C* and *D* are the mean ± SD of three experiments. ∗*p* < 0.05 using one-way ANOVA with Dunnett’s multiple comparisons test (*C* and *D*). PGO, phenylglyoxal.

We next sought to determine if DJ-1 esterase activity is also conserved in its bacterial homologs. To examine this, we expressed and purified (Fig. 2*A*) the four DJ-1 homologs (YhbO, YajL, ElbB, and HchA) encoded in the *E. coli* genome (32, 33). Esterase activity, however, was limited to only DJ-1 with none of the bacterial homologs exhibiting activity (Fig. 2*B*). To rule out the possibility that the purified DJ-1 homologs were not correctly folded and thus enzymatically inactive, we assessed the α-oxoaldehyde hydratase activity (glyoxalase III activity) of HchA as reported previously (31). To avoid previously reported buffer artifacts (34), we used phosphate-containing buffers instead of Tris-containing buffers. Moreover, enzymatic activities were eliminated by mutation in the catalytic cysteine of DJ-1 or HchA (later shown in Fig. 3), indicating that the enzymatic activities are not derived from buffer but from recombinant proteins. Hydratase activity for α-oxoaldehyde was monitored by the reduction in *A*_250_ associated with phenylglyoxal (PGO) conversion to mandelic acid (Fig. 2*C*). Clear enzymatic activity was observed for HchA (Fig. 2, *D* and *E*), which was more efficient than DJ-1 (Fig. 2*D*). Next, we measured the methylglyoxalase activity of HchA. Methylglyoxal reacts reversibly with thiols to form a hemithioacetal, which absorbance at 288 nm (*A*_288_). Since the equilibrium between hemithioacetal and methylglyoxal in the presence of thiols is rapid, the *A*_288_ rapidly disappears when methylglyoxal is converted to lactate by HchA (16, 19) (Fig. 2*F*). Therefore, we incubated methylglyoxal with N-acetyl-cysteine to form a hemithioacetal before adding HchA to the reaction. As before, HchA methylglyoxalase activity was more efficient than that of DJ-1 (Fig. 2, *G* and *H*). These results ruled out the possibility that the *E. coli* DJ-1 homologs were incorrectly folded and indicate that esterase activity is not conserved in the DJ-1 homologs. Taken together, these results conclusively demonstrate that DJ-1 has not oxidation-dependent but conventional esterase activity and that the esterase activity is not conserved in prokaryotic homologs of DJ-1.

Although DJ-1 and HchA both possess α-oxoaldehyde hydratase activity, only DJ-1 can function as an esterase (Fig. 2). To identify the amino acid residues in DJ-1 and HchA responsible for these enzymatic differences, we used the available structural information for both enzymes to introduce multiple mutations into their respective catalytic centers. Similar to the cysteine protease catalytic triad, HchA utilizes a similar arrangement of the Cys (nucleophile), His (base), and Glu/Asp (acid) residues to form a triad consisting of C185, H186, and D214, the latter of which is provided in *trans* as part of the dimeric HchA complex (Fig. 3*A*, left). The HchA H186 position, however, is not conserved in DJ-1 and as such the catalytic triad is not formed with the nucleophilic C106 (Fig. 3*A*, right). Although H126 in DJ-1 has been suggested as an equivalent residue, its imidazole ring is not oriented to function as the catalytic triad base (12, 15) (Fig. 3*A*, blue). Further, the D214 position is not conserved in DJ-1, because the equivalent position—D131—adopts a completely different orientation that precludes precise alignment of the respective ternary structures (Fig. 3*A*, green).

**Figure 4:**
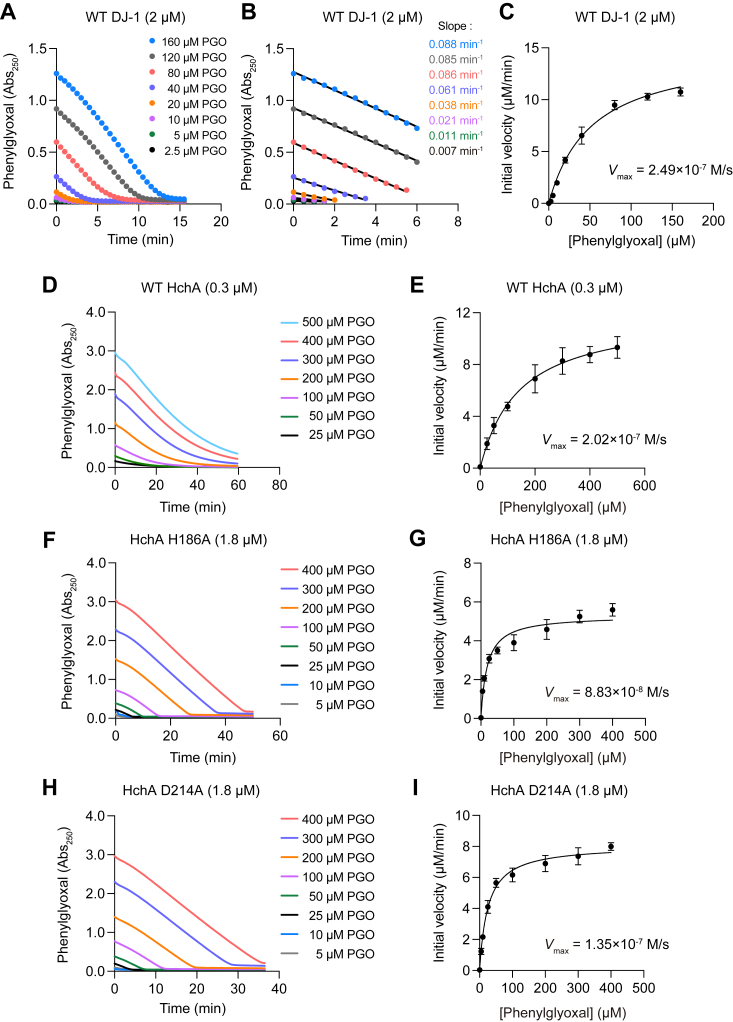
**Phenylglyoxalase enzyme kinetics of HchA H186A and HchA D214A**. *A*, PGO consumption over time by 2 μM WT DJ-1. The substrate concentrations assayed are indicated. *B*, initial velocities of PGO consumption. Each reaction velocity was calculated *via* linear regression of the data shown in *A*. *C*, Michaelis–Menten plot of WT DJ-1–mediated PGO consumption. Values were obtained from the reaction velocities shown in *B*. The *V*_max_ was calculated as 2.49 × 10^−7^ M/s. *D*, PGO consumption over time by 0.3 μM WT HchA. The substrate concentrations assayed are indicated. *E*, Michaelis–Menten plot of WT HchA-mediated PGO consumption. The *V*_max_ was calculated as 2.02 × 10^-7^ M/s. *F*, PGO consumption over time by 1.8 μM HchA(H186A). The substrate concentrations assayed are indicated. *G*, Michaelis–Menten plot of HchA(H186A)-mediated PGO consumption. The *V*_max_ was calculated as 8.83 × 10^−8^ M/s. *H*, PGO consumption over time by 1.8 μM HchA(D214A). The substrate concentrations assayed are indicated. *I*, Michaelis–Menten plot of HchA(D214A)-mediated PGO consumption. The *V*_max_ was calculated as 1.35 × 10^−7^ M/s. The data shown in *A*, *B*, *D*, *F*, and *H* are representative data of three individual experiments. Data in *C*, *E*, *G*, and *I* are the mean ± SD of three replicates. PGO, phenylglyoxal.

In contrast, the Glu residues that border the active center Cys along the opposite side of the His base are well conserved in DJ-1 (E18) and HchA (E77) (Fig. 3*A*, magenta). In addition, a GlyGly motif (G74 and G75 in DJ-1; G153 and G154 in HchA) that may form an oxyanion hole is highly conserved near the active center of both enzymes (Fig. 3*A*, orange). Based on this structural data, we generated three catalytic site mutants (C185S, H186A, and D214A) and three additional mutants (E77A, G153S, and G154S) targeting sites surrounding HchA C185 that are mechanistically interesting. After purification (Fig. 3*B*), the respective phenylglyoxalase and methylglyoxalase activities were examined (Fig. 2, *C* and *F*). When WT HchA was incubated with PGO, there was a rapid reduction in *A*_250_, whereas no decrease was observed for the E77A, G153S, G154S, and C185S mutants (Fig. 3, *C* and *E*), indicating that these residues are essential for phenylglyoxalase activity. In contrast, the H186A and D214 mutations had a little effect on phenylglyoxalase activity (t = 30 min) (Fig. 3*C*). Similarly, methylglyoxalase activities comparable to WT were observed in HchA H186A and D214A mutants following 120 min incubation (Fig. 3*D*). Therefore, the H186 and D214 residues in HchA are not essential for α-oxoaldehyde hydratase activity of two substrates (PGO and methylglyoxal) in the long-time endpoint assay; the importance of these residues in regulating the fine tuning of HchA enzymatic activity will be discussed later in Figure 4. Meanwhile, the loss of both phenylglyoxalase and methylglyoxalase activities with the C185S, E77A, G153S, and G154S mutants (Fig. 3, *C*–*E*) suggests that C185 comprises the catalytic center of HchA and that the surrounding amino acid residues (E77, G153, and G154) are enzymatically important. Aspects of these results are consistent with previous reports (31).

To more fully characterize the HchA mutants, we quantitatively measured the enzymatic kinetics of the phenylglyoxalase reaction in relation to WT HchA and DJ-1 (Fig. 4). Different concentrations of PGO were incubated with WT DJ-1 (Fig. 4*A*), WT HchA (Fig. 4*D*), HchA H186A (Fig. 4*F*), and HchA D214A (Fig. 4*H*). The reduction in *A*_250_ over time was then used to determine the initial velocities of each substrate concentration (Fig. 4*B*) with Michaelis–Menten plots generated accordingly for WT DJ-1 (Fig. 4*C*), WT HchA (Fig. 4*E*), HchA H186A (Fig. 4*G*), and HchA D214A (Fig. 4*I*). The *V*_max_ and *K*_m_ (Table 1) were determined by curve fitting against Michaelis–Menten plots and the *k*_cat_ (Table 1) of each protein was calculated as *V*_max_/[E]_0_. The HchA *K*_m_ (∼150 μM) for PGO is about three times higher than that of DJ-1 (∼50 μM), indicating lower substrate affinity. However, the HchA *k*_cat_ (∼40 min^−1^) is about six times higher than that of DJ-1 (∼7 min^−1^), indicating faster substrate conversion by HchA than DJ-1. Further, the *k*_cat_/*K*_m_ of DJ-1 (∼2400) is about half that of HchA (∼4700). Thus, as a phenylglyoxalase, HchA is more efficient than DJ-1. Next, we tried to determine if the H186A mutation affected the phenylglyoxalase activity of HchA. Comparable *K*_m_ values for the HchA D214A (∼26 μM) and H186A (∼19 μM) mutants suggest increased substrate affinity compared to WT HchA. In contrast, the *k*_cat_ of HchA D214A (∼4.5 min^−1^) and H186A (∼3 min^−1^) were lower than that of WT HchA and the *k*_cat_/*K*_m_ values for HchA D214A (∼3000) and H186A (∼2600) indicate catalytic efficiencies that are 55% and 64% of WT HchA. These results clearly demonstrate that mutations to either His or Asp in the putative HchA catalytic triad [Cys-His(base)-Asp(acid)] do not completely eliminate enzymatic activity, but do affect substrate affinity and turnover. Although the kinetic values have slight differences, our conclusion is basically consistent with several prior reports (31, 35, 36, 37).

**Table 1:** Kinetics of DJ-1 and HchA exhibiting phenylglyoxalase activity

Protein name	*K*_m_ m (M)	*k*_cat_ (s^−1^)	*k*_cat_/*K*_m_ (s^−1^ M^−1^)
WT DJ-1	5.22 ± 0.65 × 10^−5^	0.12 ± 0.007	2396 ± 228.7
DJ-1 (A107H)	6.44 ± 1.13 × 10^−4^	1.65 ± 0.127	2598 ± 302.7
WT HchA	1.49 ± 0.40 × 10^−4^	0.67 ± 0.053	4729 ± 1253
HchA (H186A)	1.87 ± 0.24 × 10^−5^	4.91 ± 0.40 × 10^−2^	2642 ± 201.5
HchA (D214A)	2.55 ± 0.47 × 10^−5^	7.50 ± 0.50 × 10^−2^	2984 ± 354.9

**Figure 5:**
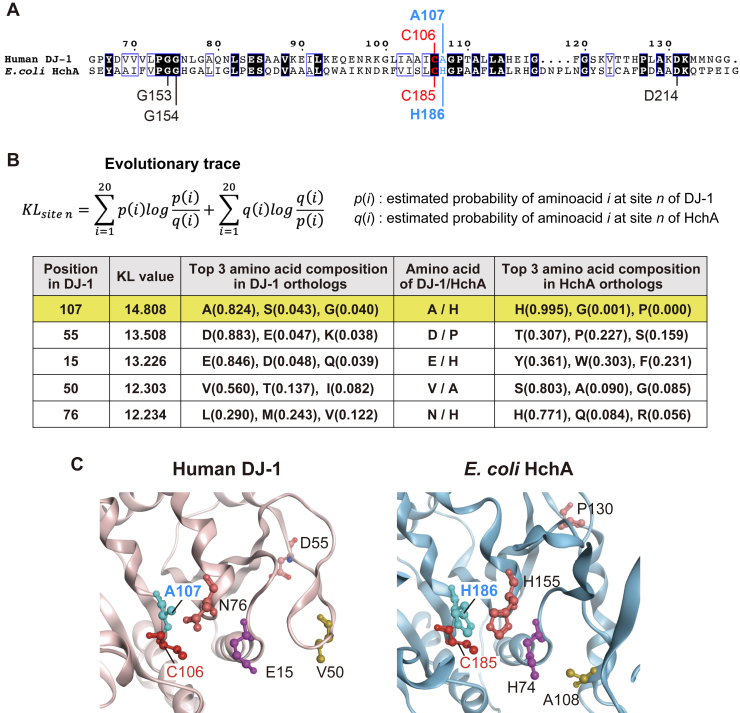
**Detection of amino acid sites involved in functional differentiation between DJ-1 orthologs and HchA orthologs.***A*, sequence alignment between human DJ-1 and *Escherichia coli* HchA. The alignment was generated using Clustal W and ESPript3 (http://espript.ibcp.fr). DJ-1 A107 is comparable to His186 in HchA (shown in *blue font*). Identical amino acids are shown in *black*, conserved sequences in *blue boxes*, catalytic cysteines in *red*, and amino acids mutated in HchA are indicated. *B* and *C*, top five sites with high KL values (*B*) and the amino acid residues with high KL values are depicted on the structures of human DJ-1 (PDB ID: 1P5F) and *E. coli* HchA (PDB ID: 1N57) (*C*). Structural visualization of DJ-1 and HchA was performed using the Molecular Operating Environment software (MOE 2020.0901) (Chemical Computing Group ULC, 910-1010 Sherbrooke St. W., Montreal, QC H3A 2R7, Canada, 2024.). KL, Kullback–Leibler; PGO, phenylglyoxal.

**Figure 6:**
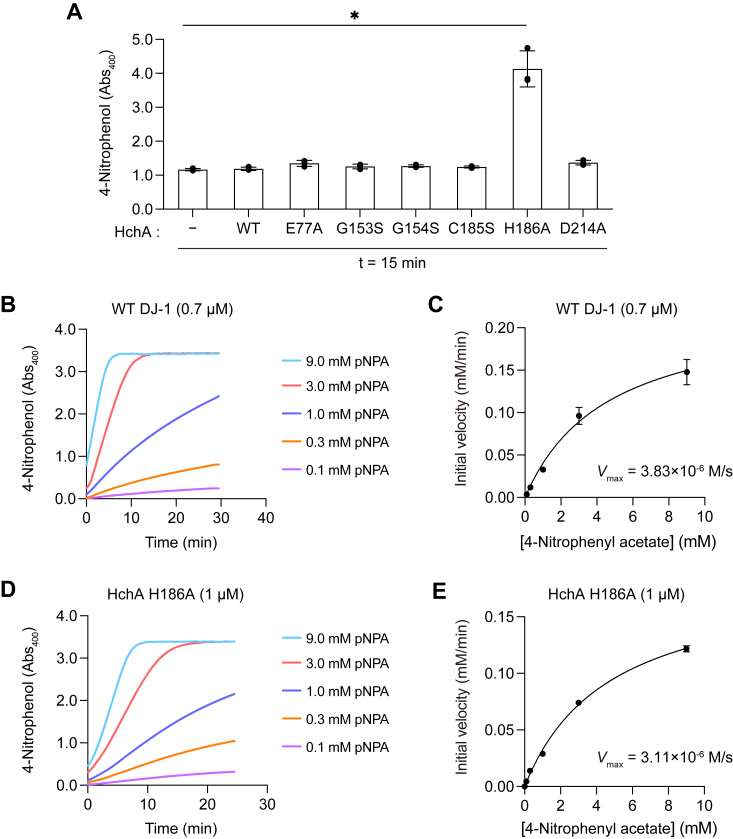
**Latent esterase activity of HchA is induced by the H186A mutation.***A*, esterase activity in WT HchA or the indicated HchA mutants. pNPA and the indicated recombinant protein were incubated as described in the experimental procedures for 15 min. *B*, formation of pNP over time by 0.7 μM WT DJ-1. The substrate concentrations assayed are indicated. *C*, Michaelis–Menten plot for WT DJ-1–mediated formation of pNP. The *V*_max_ was calculated as 3.83 × 10^−6^ M/s. *D*, formation of pNP over time by 1 μM HchA H186A. The substrate concentrations assayed are indicated. *E*, Michaelis–Menten plot for HchA(H186A)-mediated formation of pNP. The *V*_max_ was calculated as 3.11 × 10^−6^ M/s. The data shown in *B* and *D* are representative of three individual experiments. Data in *A*, *C*, and *E* are the mean ± SD of three replicates. pNP, 4-nitrophenol; pNPA, 4-nitrophenyl acetate.

Similar characterization of the esterase activity in the HchA mutants yielded interesting insights into evolutionary functional differentiation. The esterase activity was limited to DJ-1, as none of the *E. coli* DJ-1 homologs such as HchA was capable of pNPA hydrolysis (Fig. 2*B*) whereas DJ-1 function as both phenylglyoxalase and methylglyoxalase, similar to HchA. Those observations suggests that DJ-1 acquired the esterase activity as a consequence of functional differentiation in the evolutionary path to DJ-1 or all *E. coli* homologs of DJ-1 including HchA lost the esterase activity in the evolutionary path to them. Thus, we performed a modified evolutionary trace analysis (38) based on difference of amino acid composition between the DJ-1 and HchA orthologs to estimate sites relating to functional differentiation on DJ-1 and HchA. The evolutionary trace analysis estimated A107 (DJ-1)/H186 (HchA) as the most relevant site in functional differentiation (Fig. 5, *A* and *B*); the conservation of A107 (DJ-1) and H186 (HchA) are 82% and 99%, respectively. Interestingly, the site locates close to the catalytic cysteine of DJ-1 and HchA (Fig. 5, *A* and *C*). We therefore hypothesized that the mutation of this site is closely related to the acquisition of the esterase activity and measured the esterase activity of the HchA H186A mutant. The mutant expectedly showed clear esterase activity (Fig. 6*A*). As before, initial velocities were determined by reacting different concentrations of pNPA with WT DJ-1 (Fig. 6*B*) and HchA H186A (Fig. 6*D*). Michaelis–Menten plots (Fig. 6, *C* and *D*) were used to determine the *V*_max_, *K*_m_, and *k*_cat_ (Table 2). Although DJ-1 and HchA H186A had comparable *K*_m_ values (∼4.8 mM), the DJ-1 *k*_cat_ (330 min^−1^) was higher than the HchA H186A *k*_cat_ (187 min^−1^). Consequently, the DJ-1 *k*_cat_/*K*_m_ was 1.8 times higher than the HchA H186A mutant. Further, while the gain in esterase activity by the HchA H186A mutant was modest (∼55% in *k*_cat_/*K*_m_) relative to DJ-1, it is clear that the activity, which had been absent in the WT HchA, emerged following introduction of the H186A mutation.

**Table 2:** Kinetics of WT DJ-1 and the HchA H186A mutant exhibiting esterase activity

Protein name	*K*_m_ (M)	*k*_cat_ (s^−1^)	*k*_cat_/*K*_m_ (s^−1^ M^−1^)
WT DJ-1	4.76 ± 1.25 × 10^–3^	5.47 ± 0.935	1178 ± 203.2
HchA (H186A)	4.76 ± 0.36 × 10^–3^	3.11 ± 0.144	655 ± 19.42

**Figure 7:**
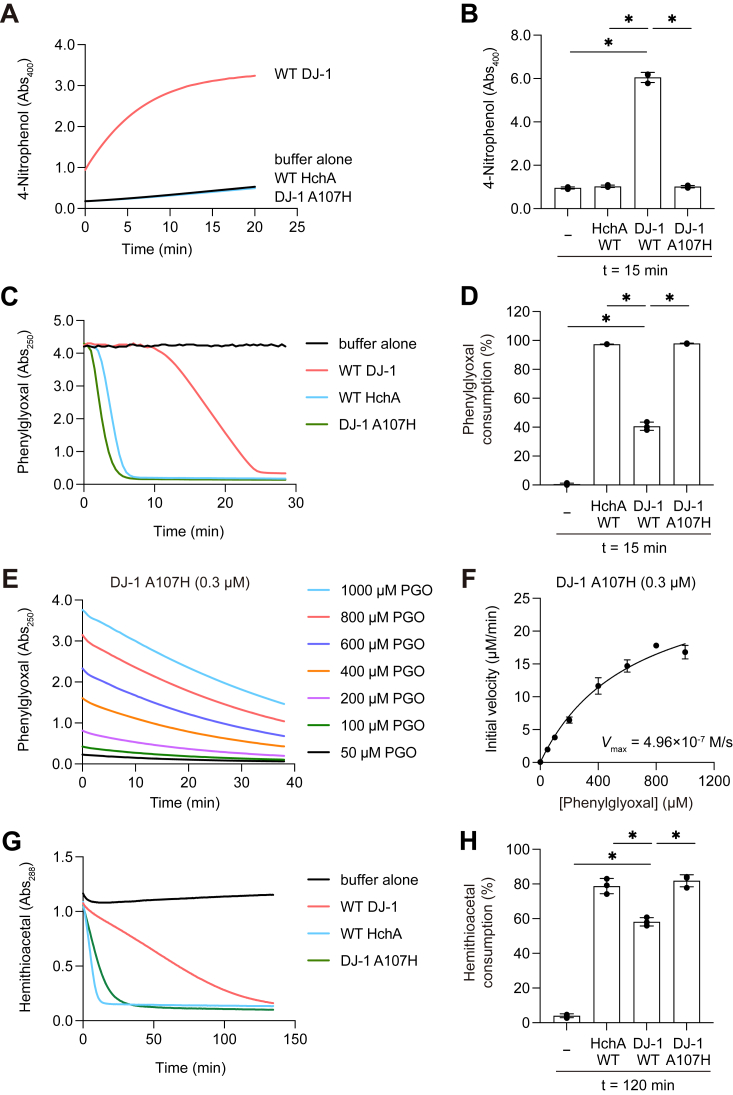
**The A107H mutation autoinhibits DJ-1 esterase activity.***A*, production of 4-nitrophenol (pNP) over time. Indicated proteins and 4-nitrophenyl acetate (pNPA) were incubated as described in the Experimental procedures. *B*, inhibition of pNP formation by the DJ-1 A107H mutation. The enzymatic reactions were incubated for 15 min. *C*, reduction in PGO-dependent *A*_250_ over time. PGO was incubated with WT DJ-1 (*red*), WT HchA (*blue*), or the DJ-1(A107H) mutant (*green*) as described in the experimental procedures. The *black line* indicates the buffer control. *D*, PGO consumption over time. PGO was incubated for 15 min with the indicated proteins (all n = 3). *E*, decrease in PGO by 0.3 μM DJ-1(A107H). The substrate concentrations assayed are indicated. Initial velocities are based on the data shown. *F*, Michaelis–Menten plot for DJ-1(A107H)-mediated consumption of PGO (n = 3). The *V*_max_ was calculated as 4.96 × 10^−7^ M/s. *G*, DJ-1(A107H)-mediated hemithioacetal consumption. Reaction conditions for buffer alone (*black*), WT DJ-1 (*red*), WT HchA (*blue*), and the DJ-1 A107H mutant (*green*) are as described in the Experimental procedures. *H*, hemithioacetal consumption following 120 min incubation with the indicated proteins (all n = 3). Data shown in *A*, *C*, and *G* are representative of three individual experiments. Data in *B*, *D*, and *H* are the mean ± SD of three experiments. PGO, phenylglyoxal.

To establish the importance of this site in functional differentiation, we also tested whether the reciprocal A107H mutation would disrupt esterase activity in DJ-1. We generated and purified a DJ-1 A107H mutant and examined its esterase activity. As shown in Figure 7, *A* and *B*, we found that the DJ-1 A107H mutation almost completely abolished the esterase activity. In contrast, the phenylglyoxalase activity in the mutant was enhanced relative to WT (Fig. 7, *C* and *D*). To confirm this result, we determined initial velocities (Fig. 7*E*) and generated Michaelis–Menten plots (Fig. 7*F*) to obtain the associated *V*_max_, *K*_m_, and *k*_cat_ for the DJ-1 A107H mutant (Table 1). Even though the PGO *K*_m_ (644 μM) of the mutant was ∼12-fold higher than WT DJ-1 (52 μM), indicating weakened affinity for the substrate, the A107H mutation dramatically enhanced the *k*_cat_ (100 min^−1^) to ∼14-fold higher than WT DJ-1 (7.2 min^−1^) (Table 1). These values suggest that conversion of PGO to mandelic acid was substantially accelerated by the A107H mutation when a sufficient amount of substrate was present in the experimental conditions (Fig. 7*C*). Similarly, the methylglyoxalase activity (Fig. 7, *G* and *H*) of DJ-1 was also accelerated by the A107H mutation. These results indicate that a mutation in the amino acid adjacent to the catalytic center of DJ-1 family proteins can regulate the presence or absence of their esterase activity and suggest the possibility that DJ-1 esterase activity was acquired evolutionarily through such a mutation.

## Discussion

We have presented multiple lines of evidence supporting three significant conclusions regarding DJ-1 esterase activity. Our first conclusion, that DJ-1 exhibits oxidation-independent esterase activity *in vitro*, is supported by significant reductions in hydrolysis of pNPA following hydrogen peroxide-mediated oxidation of the active site Cys (C106). This result indicates that previously reported oxidation-activated esterase activity for DJ-1 (23) was not reproducible. C106 is the nucleophile for DJ-1 enzyme reaction as well as the target of oxidation, thus it was unclear how oxidative modification of C106 could enhance esterase activity. We think that not oxidation-dependent but oxidation-independent/inhibited esterase activity of DJ-1 is biochemically reasonable. Our second conclusion, that esterase activity is not evolutionarily conserved in *E. coli* homologs of DJ-1, was developed from our use of 4 *E. coli* DJ-1 homologs (YajL, YhbO, ElbB, or HchA) to evaluate DJ-1 enzymatic activities. While the bacterial homologs, in particular HchA, exhibited some of the functionalities reported for DJ-1, including α-oxoaldehyde hydratase activity, which converts α-oxoaldehyde to α-keto-acid, none of the homologs exhibited esterase activity.

Our third, and most significant, conclusion regarding the evolutionary origin of the esterase activity is derived from the targeted mutations we generated in HchA and DJ-1. While HchA does not naturally exhibit esterase activity, the introduction of the H186A mutation at the residue adjacent to the nucleophilic Cys based on evolutionary trace analysis resulted in HchA gaining esterase activity (Fig. 6*A*). Furthermore, insertion of the opposite A-to-H mutation at A107, the site in DJ-1 equivalent to HchA H186A, abolished esterase activity (Fig. 7, *A* and *B*). Although this site is adjacent to the active Cys residue (C106 in DJ-1), the mutation effects were specific to the esterase function as α-oxoaldehyde hydratase activity was enhanced rather than inhibited (Fig. 7, *C* and *E*). Given these findings, we were curious as to why the HchA H186A mutant acquired esterase activity, whereas the DJ-1A107H mutant specifically lost esterase activity. By comparing the reaction kinetics for conversion of a PGO substrate (Tables 1 and 2), we were able to develop a potential molecular mechanism for the reaction. The insertion of the A-to-H mutation near the DJ-1 catalytic Cys resulted in an ∼12-fold increase in *K*_m_, indicating a significant decrease in substrate affinity. Despite this, the enzymatic *k*_cat_/*K*_m_ slightly increased, which is attributable to a substantial improvement (∼14-fold increase) in *k*_cat_ by the A-to-H mutation. Conversely, the H-to-A mutation in the same region of HchA resulted in a ∼12% reduction in *K*_m_, which corresponds to increased substrate affinity. However, similar to DJ-1, the mutation also decreased *k*_cat_ (reducing *k*_cat_ to about 7%), ultimately resulting in reduced enzymatic activity (inferred from *k*_cat_/*K*_m_). In either case, having Ala adjacent to the catalytic Cys [corresponding to WT DJ-1 and the HchA H186A mutant] leads to a smaller *K*_m_ and higher substrate affinity. As a result, the affinity for pNPA is enhanced by the H186A mutation in HchA, and esterase activity proceeds *in vitro*. Conversely, if the residue adjacent to the active center Cys is His [corresponding to WT HchA and the DJ-1 A107H mutant], the *K*_m_ increases and substrate affinity decreases. Under these conditions, pNPA was not recognized by the DJ-1 A107H mutant due to the elevated *K*_m_ and esterase activity was lost. However, if this is the case, the presence of the A107H mutation in DJ-1 should increase the *K*_m_ (*i.e.*, decrease affinity) for PGO and phenylglyoxalase activity should likewise be inhibited. The *K*_m_ of DJ-1 for PGO (5.2 × 10^−5^ M) is two orders of magnitude lower than the *K*_m_ for pNPA (4.7 × 10^−3^ M). Consequently, even though the A107H mutation resulted in an ∼12-fold increase in the *K*_m_ compared to WT DJ-1 (Table 1), the 640 μM *k*_m_ in the mutant was still sufficient to allow for PGO binding and recognition as a substrate. When substrate recognition occurs, the enzymatic activity of the DJ-1A107H mutant should be apparent as an increase in *k*_cat_. In effect, the difference in *K*_m_ led to selective inhibition of the esterase activity with narrowed substrate specificity in the DJ-1 A107H mutant, whereas expanded substrate specificity in the HchA H186A mutant resulted in the emergence of esterase activity. These findings suggest that esterase activity arose from a missense mutation near the catalytic Cys in the DJ-1 ancestral protein.

Given that DJ-1 is implicated in antioxidative stress responses, the report of its oxidation-dependent esterase activity (23) attracted initial interest. Further, since the esterase activity of DJ-1 is easily detectable, it has been useful for identifying and characterizing DJ-1 inhibitors (24). Despite these research contexts, specific details regarding oxidation-dependent esterase activity and its evolutionary conservation remained largely unexplored. As described above, we found that DJ-1 is not oxidation-activated esterase. Moreover, we found a critical amino acid residue for DJ-1 to determine esterase activity. From an evolutionary perspective, it is interesting that a single mutation at the site (DJ-1 A107/HchA H186) estimated by the evolutionary trace analysis between DJ-1 and its bacterial homolog HchA leads to gain or lack of esterase activity. Even though we hypothesized that esterase activity arose from a missense mutation near the catalytic Cys in the DJ-1 ancestral protein, YajL, a close *E. coli* homolog of DJ-1 that also has an Ala following the active site Cys, shows no esterase activity (Fig. 2*B*). We surmise that YajL probably has some additional mutation that weakens general enzymatic activities including esterase activity as YajL possesses neither esterase (this study) nor methylglyoxalase activity (33). Regarding the esterase activity of DJ-1, what is the physiological significance of the activity we examined? Recently, cyclic 3-phosphoglyceric anhydride (cPGA) has attracted attention as a novel substrate for DJ-1 (11, 39). It is worth noting that the cPGA is a highly reactive cyclic ester (*i.e.*, reactive lactone), and DJ-1 has been suggested to hydrolyze the ester bond of cPGA to detoxify it. Therefore, if the cPGA is a physiological substrate for DJ-1, the esterase activity we examined might reflect its physiological enzymatic function.

We generated phylogenetic profiles of DJ-1 and its bacterial homolog and analyzed the conservation or loss of these genes in the eukaryotic lineage (Fig. S1). DJ-1/YajL was conserved whereas YhbO and HchA were lost in human and relates species. Ancestral DJ-1 might obtain esterase activity in divergence to eukaryotes because of the lack of esterase activity of bacterial homologs and then acquire methylglyoxalase activity to compensate for the loss of YhbO and HchA genes in path to human and related species. This study indicates that mutations around the active center of DJ-1 and its homologous protein can change the substrate specificity and thus can determine the enzymatic character of DJ-1 homologs during evolution.

## Experimental procedures

### Plasmids

The HchA, YajL, YhbO, and ElbB genes were PCR amplified from an *E. coli* genomic DNA template. PCR fragments were cloned by SLiCE into the pET21a vector to obtain N-terminal His-tag expression plasmids for HchA, YajL, YhbO, and ElbB. The HchA mutant plasmids were constructed using a classical two-step PCR method. DJ-1 expression plasmids with an N-terminal His-tag were constructed by cloning DJ-1 into the pET28a vector as reported previously (40). C106D or A107H mutagenesis of DJ-1 was carried out using the quick-change method. The mutations were confirmed by Sanger DNA sequencing.

### Purification of recombinant proteins

Expression plasmids for DJ-1, HchA, YajL, YhbO, and ElbB were transformed into BL21 (DE3) + RIL (Agilent Technologies). A single colony was inoculated into 2 ml LB media containing 25 μg/ml of kanamycin or 100 μg/ml of ampicillin and cultured overnight at 37 °C and 170 rpm. The preculture was added to 100 ml LB and cultured for 2 h at 32 °C and 120 rpm. Once the culture reached an absorbance of 0.3 to 0.5 at 600 nm, 0.3 mM IPTG was added and cultured for another 3 h at 32 °C and 120 rpm. Bacteria were collected by centrifugation at 5800*g* for 10 min. Pellets were resuspended in lysis buffer [20 mM Tris buffer (pH 7.5), 200 mM NaCl, 10 mM *β*-mercaptoethanol, 1 μg/ml lysozyme (Wako), 1 μg/ml DNase I, and 5 mM MgCl2] and sheared using a sonicator. Cellular debris was removed by centrifugation at 5800*g* for 10 min at 4 °C and the resulting supernatant recovered. His-tagged recombinant proteins were purified using standard procedures with nickel-agarose (Ni-NTA Agarose, Qiagen) and associated elution buffers (200 mM NaCl, 10 mM *β*-mercaptoethanol, and 250–500 mM imidazole in 20 mM Tris buffer pH 7.5). To remove the imidazole, the eluted samples were dialyzed twice against 1 L of dialysis buffer (200 mM NaCl and 1 mM DTT in 20 mM Tris buffer pH 7.5) for at least 4 h using 10 K molecular weight cut-off cassettes (Thermo Fisher Scientific). Proteins purity was confirmed by Coomassie Brilliant Blue–stained SDS-PAGE gels. Protein concentrations were determined using a BCA protein assay kit (Pierce). Structures of the putative HchA catalytic center (PDB code: 1N57) and human DJ-1 (PDB code: 1P5F) were drawn with Chimera (www.cgl.ucsf.edu/chimera).

### Measurement of esterase activity

The esterase assay used pNPA (Sigma-Aldrich) as the substrate and hydrolysis was detected by measuring the absorbance of pNP at 400 nm (*A*_400_) as described previously (23). In brief, 1 μM recombinant protein (DJ-1, YajL, HchA, YhbO, ElbB, DJ-1 C106D, DJ-1 A107H, HchA E77A, HchA G153S, HchA G154S, HchA C185S, HchA H186A, and HchA D214A) and 1.6 mM pNPA were incubated in 50 mM sodium phosphate buffer (pH 7.0) at 37 °C. *A*_400_ was monitored over time using a spectrophotometer (Scrum) and an Enspire plate reader (PerkinElmer). To determine if oxidation affected DJ-1 esterase activity, WT DJ-1 or the C106D mutant was pre-incubated with 400 μM H_2_O_2_ for 1 h at room temperature prior to reacting with pNPA.

### Measurement of phenylglyoxalase and methylgloxalase activity

PGO has an advantage as a mimetic substrate for the putative physiological substrate, methylglyoxal. Specifically, changes in PGO levels can be monitored as changes in absorbance, allowing for real-time monitoring of substrate consumption without affecting the experimental system. This advantage makes PGO an ideal model substrate to evaluate α-oxoaldehyde hydratase activity of DJ-1. To assess phenylglyoxalase activity, 5 μM purified protein (WT DJ-1, DJ-1 A107H, WT HchA, or the following HchA mutants—E77A, G153S, G154S, C185S, H186A, and D214A) was incubated with 1 mM PGO (TCI Chemicals) in 20 mM sodium phosphate buffer (pH 7.0) at 37 °C. The consumption of PGO was monitored by a reduction in absorbance at *A*_250_ over time (41). To assess methylglyoxalase activity, 7.5 mM methylglyoxal (Sigma-Aldrich) and 7.5 mM N-acetyl-L-cysteine (Sigma-Aldrich) were preincubated at room temperature for 30 min in 50 mM sodium phosphate buffer (pH 7.0) to form a hemithioacetal. Recombinant protein (5 μM) was then added to the reaction mixture as previously reported (40). Hemithioacetal levels were monitored over time by measuring absorbance at *A*_288_. Both assays were conducted using a spectrophotometer and an Enspire plate reader.

### Quantification of enzymatic parameters

Each purified protein was mixed with 5 to 8 different concentrations of PGO or pNPA and changes in *A*_250_ or *A*_400_ over time were measured. The initial velocities were obtained *via* linear regression of the resulting data. To calculate *V*_max_ and *K*_m_, the initial velocity data were fitted to a Michaelis–Menten plot using Prism 10 (GraphPad Software). The catalytic constant *k*_cat_ is equal to *V*_max_ divided by the purified protein concentration. All measurements were repeated at least three times and either representative data or average values are reported.

### Detection of amino acid sites involved in functional differentiation between DJ-1 and HchA

Amino acid sequences of 27 DJ-1 orthologs and 24 HchA orthologs that share sequence identity <40% were collected from the OrthoMCL DB (42). Then a multiple sequence alignment that included all of two ortholog groups was generated using MAFFT (43). The difference between the DJ-1 and HchA orthologs in the amino acid composition at each alignment site was evaluated by our previously developed evolutionary trace analysis (38) based on the Kullback–Leibler (KL) symmetric divergence value. The KL value is calculated as follows:

$${KL}_{site\;n}=\sum_{i=1}^{20}p\left(i\right)\log\frac{p\left(i\right)}{q\left(i\right)}+\sum_{i=1}^{20}q\left(i\right)\log\frac{q\left(i\right)}{p\left(i\right)}$$

where *p*(*i*) and *q*(*i*) are the probability of amino acid *i* at an alignment site n of the DJ-1 and HchA orthologs, respectively. The KL values were used to predict the sites involved in the functional differentiation between DJ-1 and HchA.

### Statistical analysis

All data are presented as means  ±  SD, unless otherwise indicated. Statistical significance was determined using either one-way or two-way ANOVA with Bonferroni’s or Dunnett’s multiple comparison test. Significance values are indicated as ∗*p*  <  0.05. All statistical analyses were performed using Prism 10.

## Data availability

The data generated are included in the main text file.

## Supporting information

This article contains supporting information.

## Conflict of interest

The authors declare that they have no conflicts of interests with the contents of this article.
