# Supplementary material for: The origin of esterase activity of Parkinson's disease causative factor DJ-1 implied by evolutionary trace analysis of its prokaryotic homolog HchA
Source: J Biol Chem. 2024 Jun 13;300(7):107476. doi: 10.1016/j.jbc.2024.107476 (PMC11301059; doi:10.1016/j.jbc.2024.107476)
Supplement: Supplementary materials [file mmc1.pdf]

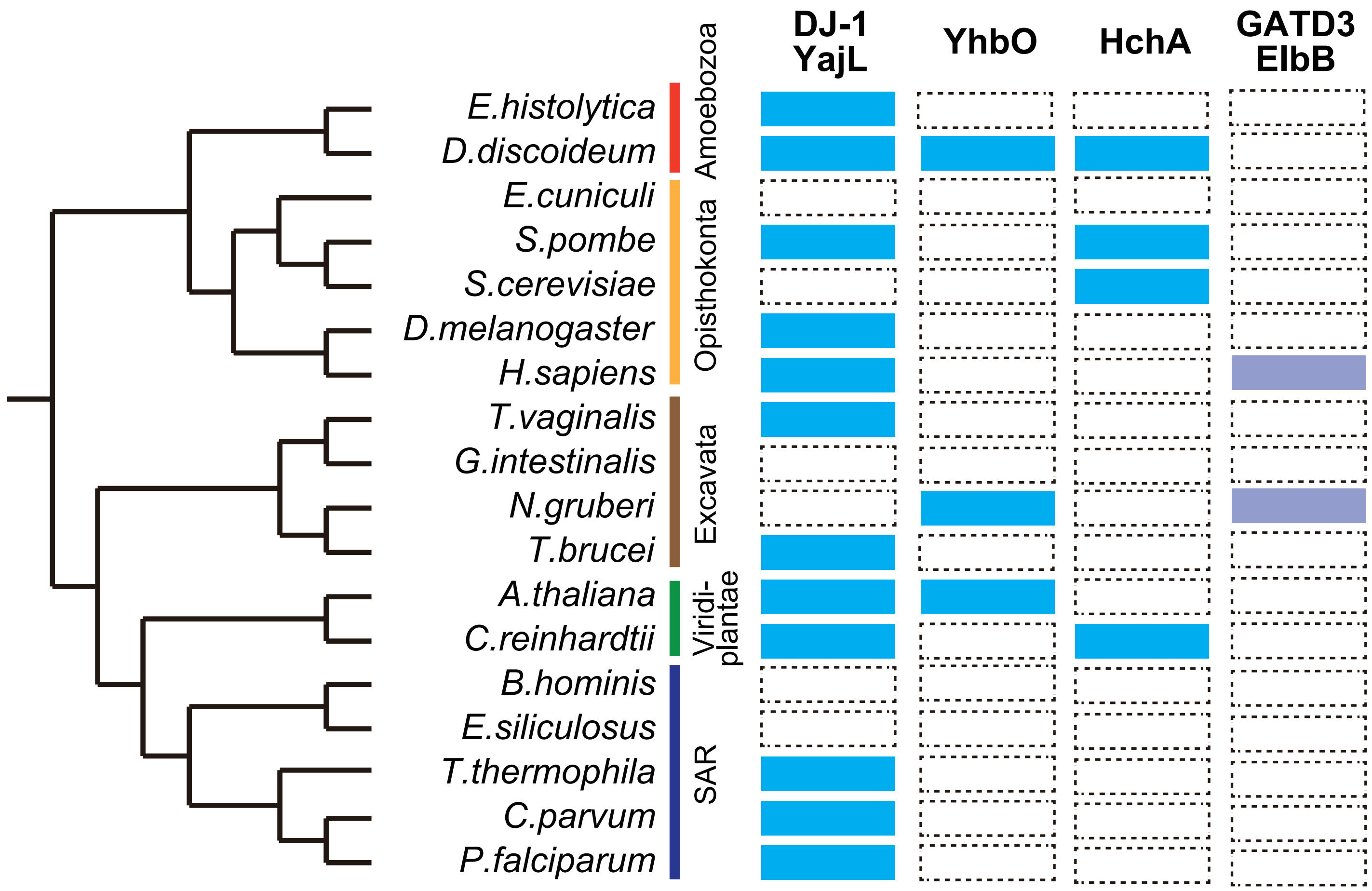

Fig. S1. Evolutionary profile for orthologs of *E.coli* DJ-1 homologs in eukaryotic lineage

Evolutionary profile was generated using ortholog information obtained from OrthoMCL DB (https://orthomcl.org/orthomcl/app). Although ElbB shows sequence similarity to human DJ-1 and *E. coli* YajL, YhbO, and HchA, it has more significant sequence similarity to human GATD3.
